# Effects of inclusion level and amino acid supplementation on energy values of soybean oil determined with difference or regression methods in growing pigs

**DOI:** 10.5713/ajas.18.0944

**Published:** 2019-04-15

**Authors:** Qiuyun Wang, Chengfei Huang, Mei Liu, Ling Liu, Shuai Zhang

**Affiliations:** 1 State Key Laboratory of Animal Nutrition, Ministry of Agriculture Feed Industry Centre, China Agricultural University, Beijing 100193, China; 2 Milk and Dairy Product Inspection Center of the Ministry of Agriculture, Beijing 100193, China

**Keywords:** Amino Acid, Difference Method, Energy, Regression Method, Soybean Oil

## Abstract

**Objective:**

This study was conducted to evaluate the effects of inclusion level and amino acid (AA) supplementation on energy values of soybean oil (SO) as determined by difference method or regression method when fed to growing pigs.

**Methods:**

Thirty-six barrows (initial body weight: 28.0±1.3 kg) were randomly assigned to one of 6 dietary treatments, which included 2 control diets formulated using a basal diet with or without AA supplementation, and 4 experimental diets with 5% or 10% SO addition in the 2 control diets, respectively. All pigs were individually housed in metabolism crates for 19 d, and during the last 5 d, total urine and feces production were collected. The nutrient digestibility in diets and the digestible energy (DE) and metabolizable energy (ME) values of SO were determined using the difference method and the regression method, respectively.

**Results:**

Our results showed that there were no interaction effects (p>0.05) between AA supplementation and SO inclusion levels on energy values of SO and dietary nutrient digestibility. The DE and ME values of SO determined by the difference method were not affected (p>0.05) by AA supplementation, however, the ME value of SO increased (p<0.05) as the inclusion level of SO increased. Moreover, the energy values of SO determined using the regression method were close to those determined using difference method with 10% SO inclusion, but were greater than those obtained using difference method with 5% SO inclusion.

**Conclusion:**

We concluded that the DE and ME values of SO increased with the inclusion level but were not affected by AA supplementation in the range of 0% to 10%. The difference method can substitute for the regression method to determine the DE and ME values of SO when the inclusion level is 10%, but not at 5% inclusion level.

## INTRODUCTION

Energy is the most expensive nutritional component in animal diets. As an important energy source, lipid is commonly included in swine diets to improve the growth rate and feed efficiency of pigs [1,2]. Dietary lipid supplementation can also improve the digestibility and utilization of other nutrients in diets [3]. Therefore, it is important to accurately evaluate energy values of dietary lipids. Adequate and balanced amino acid (AA) content is another vital nutritional concern in swine diets. Crystalline AA supplementation in diets can influence energy utilization, feed intake, growth performance and carcass quality of pigs [4,5], as well as, affect nitrogen deposition and utilization [6,7].

There are discrepancies in the energy value or soybean oil (SO) among different national and regional feed databases [8–11], which could be attributed to SO inclusion level, the basal diet composition, the processing technique of SO [9,12–14]. Previous work conducted by our research group reported the effect of basal diet type (corn-soybean meal diet vs. corn starch–casein diet) on digestible energy (DE) and metabolizable energy (ME) values of SO, and the results showed that the basal diet type did not affect the energy values of SO [15]. Another study explored the effects of AA supplementation and inclusion level on DE and ME value of soybean meal, and concluded that both inclusion level and AA supplementation influenced the energy values of soybean meal in pigs [16]. To our knowledge, no previous literature has reported the combined effects of inclusion level and AA supplementation on the energy value of SO.

The regression method is commonly used to evaluate the energy value of dietary lipid sources such as SO and canola oil [2,17,18]. The difference method, an indirect approach, is also used to assess the DE and ME values of feedstuff [19]. However, the lipid source inclusion level is typically limited (i.e. 5% to 10%) when using the difference method. At low inclusion levels, evaluation and efficiency would be lower than that of high inclusion level, though low inclusion level was close to actual production. As a result, the evaluation accuracy and efficiency may be influenced by the basal diet composition. To our knowledge, no studies have investigated the discrepancy between the regression and difference methods on determination of SO energy values in growing pigs especially when crystalline AA is supplied in basal diet.

Therefore, the objective of this study was to evaluate the effect of AA supplementation, SO inclusion level, and experiential method on determination of DE and ME values of SO in growing pigs.

## MATERIALS AND METHODS

The animal trial was conducted in the Metabolism Laboratory at the China Agricultural University Animal Experimental Base (Fengning, China). The protocol for the experiment was reviewed and approved by the Institutional Animal Care and Use Committee at China Agricultural University (Beijing, China).

### Animals, diets, and experimental design

Thirty-six crossbred barrows (Duron×Landrace×Yorkshire) with initial body weight (BW) of 28.0±1.3 kg were randomly assigned to one of six dietary treatments, with six replicated pigs per treatment. The dietary treatments included two control diets formulated as a corn-soybean meal basal diet with (AA+) or without (AA−) crystalline lysine, methionine, threonine, tryptophan and valine supplementation, and four experimental diets where SO was added at 5% or 10% SO in each of these two control diets. Although the inclusion level of SO in a normal diet used in the actual production is usually less than 5%, the low substitution rate would lead to high standard errors when using the difference method. That is the reason why previous studies tended to use regression method to evaluate the energy values of lipids [2,17,18]. Therefore, the higher inclusion level (5% and 10%) was used in this study to ensure a stable and accurate result, as compared with the inclusion levels used in the previous studies [9,15]. The four experimental diets were made by thoroughly mixing 5% or 10% SO with 95% or 90% basal diets (AA+ or AA−). The SO used was food-graded class four lipid and was obtained from the China Agriculture University Animal Experimental Base (Fengning, China). The energy content, AA levels, and other nutrients in the three AA+ diets were formulated to exceed the nutrient requirement for 25 kg pigs in NRC [11]. Corn replaced crystalline lysine in AA− diets. Ingredient compositions of the two control diets and the analyzed chemical compositions of the SO used in this study are listed in Table 1 and 2, respectively.

All pigs were individually housed in stainless-steel metabolism crates (1.4×0.7×0.6 m) with *ad libitum* access to water during the animal trial. The experiment lasted for 19 days, which included a 14-d period for adaptation to crates and diets, followed by a 5-d period for total feces and urine collection. Commercial diets were provided at the beginning of the experiment, and were gradually replaced by the treatment diets during the first 7-d of the adaptation period. On d 8, pigs were weighted and daily feed allotment was set at 4% of BW, in two equal meals 08:00 h and 14:00 h.

### Sample collection

During the last 5 days, total but separate collections of feces and urine were completed twice daily according to the methods described by Su et al [9]. Feces were placed into plastic bags and stored at −20°C. At the end of the collection period, the 5-d fecal production from each pig was pooled and weighed and a 350 g sub-sample was taken and dried in a forced-draft oven at 65°C for 72 h. To limit microbial growth and ammonia loss in the collected urine, approximately 50 mL of 6 N HCl was added to collection containers during the urine collection, and the urine samples were stored at −20°C immediately after collection. At the end of the collection period, total urine volume was recorded and sub-samples (4 mL) were taken and dried at 65°C for 8 h with quantitative filter paper in crucibles for energy determination.

### Chemical analysis

The fatty acid composition of SO was determined by high performance gas chromatography (6890 series, Agilent Technologies, Wilmington, DE, USA), following the method of Sukhija and Palmquist [20] with modifications. The characteristics of SO were determined according to AOCS [21] methods: p-Anisidine content was determined by AOCS Cd 18–90; the free fatty acid value was acquired by AOCS Ca 5A-40; the MIU (the sum of moisture, insoluble impurities and unsaponifiable matter) value was acquired by AOCS Ca 2c-25 (moisture), AOCS Ca 3-46 (insoluble impurities), and AOCS Ca 6a-40 (unsaponifiable matter); the peroxide value was acquired by AOCS Cd 8b-90; the hexanal value was acquired by AOCS ; the total tocopherols value was acquired by AOCS Ce 8-89. Moreover, the iodine value (IV) was calculated using the fatty acid profiles following the equation proposed by Mireia et al [22]: IV = (C16:1×0.95)+(C18:1×0.86)+(C18:2×1.732)+(C18:3×2.616)+(C20:1×0.795)+(C20:2×1.57)+ (C20:3×2.38)+(C20:4×3.19)+(C20:5×4.01)+(C22:4×2.93)+ (C22:5×3.68)+(C22:6×4.64).

All diets and fecal samples were analyzed for dry matter (DM; method 934.01, Association of Official Analytical Chemists (AOAC, 2005), acid-hydrolyzed ether extract (AEE; method 954.02, AOAC, 2005), and crude protein (CP; method 990.03, AOAC, 2005), and all urine samples were analyzed for CP (method 990.03, AOAC, 2005) [23]. In addition, the gross energy (GE) of SO, diets, feces and urine samples were measured using an Automatic Isoperibol Oxygen Bomb Calorimeter (parr 1281 Calorimeter; Parr Instrument Co., Moline, IL, USA) using benzoic acid as a standard.

### Calculation

The DE content of the diet was calculated as the difference between the total GE intake (MJ/kg of DM) and the GE content in feces (MJ/kg of DM). The ME content of the diet was calculated as the difference between the DE content in diet (MJ/kg of DM) and the GE content in urine (MJ of DM). The apparent total tract digestibility (ATTD) of GE, DM, AEE, and CP was calculated using the following equation: ATTD (%) = ([F_in_–F_out_]/F_in_)×100, where F_in_ is the nutrient component (GE, DM, AEE, or CP) in feed intake, and F_out_ is the corresponding nutrient component in feces. In the difference method, the DE and ME value of SO was calculated using the following equations: DE (MJ/kg) = (DE_diet_–DE_0_×[1–x])/x, and ME (MJ/kg) = (ME_diet_–ME_0_×[1–x])/x, where DE_diet_ and ME_diet_ are the DE and ME values of the experimental diets, DE_0_ and ME_0_ are the DE and ME values of the basal diets, and x is the inclusion levels of SO. In the regression method, the predication equations of DE and ME were established using linear regression with the inclusion ratios of 0%, 5%, and 10%. The energy values of SO were estimated using the prediction equations when x = 1.

### Statistical analysis

Data were checked for normality using the UNIVERIATE procedure of SAS (SAS Institute Inc., Cary NC, USA), and analyzed by two-way analysis of variance using general linear model procedure of SAS. The statistical model included the main effects of SO inclusion level, AA supplementation, and their interaction, with individual pig as the experimental unit. Treatment means were separated using LSMEANS statement of SAS, and were adjusted using Duncan’s multiple range test. Regression equations to estimate the DE and ME values of SO were developed using the REG procedure of SAS. The DE and ME values of SO then were estimated by solving the prediction equations when the inclusion level of SO was equal to 100%. The CLB statement of SAS was used to determine the 95% confidence interval in the regression method. Differences were considered significant at p<0.05, whereas 0.05 ≤p≤0.10 were considered statistical trends.

## RESULTS

All pigs remained healthy throughout the experiment and readily consumed their diets. Both feces and urine samples were successfully collected from all pigs.

### Energy content, nutrient digestibility, and nitrogen balance in diets

The effects of SO inclusion level and AA supplementation on energy content, nutrient digestibility and nitrogen (N) balance of experimental diets are shown in Table 3. No interaction between SO inclusion level and AA supplementation was observed for all dietary parameters measured.

Both SO inclusion and AA supplementation significantly affected GE output in urine, but did not affect GE output in feces. Dietary SO addition (5% or 10%) significantly increased GE output in urine (p = 0.01), but dietary AA supplementation significantly decreased GE output in urine (p = 0.01). The DE and ME content of the diets significantly improved as dietary SO inclusion level increased (p<0.01), but were not affected by AA addition. The ME/DE ratio increased with AA supplementation (p<0.01), meanwhile, there was a tendency for the ME/DE ratio (p = 0.07) to decrease with SO inclusion level.

There was a tendency for AA supplementation to increase ATTD of DM (p = 0.10) and AEE (p = 0.09) in diets. Moreover, the ATTD of GE, DM, OM, and AEE increased as the SO inclusion level increased from 0% to 10% (p<0.01).

For the N balance, the urinary N excretion significantly decreased (p = 0.02) and N retention significantly increased (p = 0.04) with AA addition. Additionally, fecal N excretion, N retention and ATTD of N were affected by SO inclusion levels (p = 0.04) where, 0% SO inclusion resulted in greater N retention, and 10% SO inclusion resulted in lower fecal N excretion and greater ATTD of N in diets compared to the 0% and 5% inclusion levels.

### The DE and ME values of SO determined using difference or regression method

The DE and ME values of SO determined by the difference method are shown in Table 4. No interaction between SO inclusion level and AA supplementation were observed. The ME value of SO was significantly greater when 10% SO was included in diets compared to that with 5% SO inclusion (p = 0.05). In addition, there was a tendency for the DE and ME value of SO to increase with AA supplementation (p = 0.09 and p = 0.07, respectively), while the ME/DE ratio was not affected by either SO inclusion level or AA addition.

The DE and ME values of SO determined by the regression method are shown in Table 5. With AA supplementation, the estimated intercept and slope of the prediction equations for DE and ME were 14.320 and 23.700, and 14.169 and 23.000 MJ/kg, respectively (p<0.01), and the coefficients of determination (R^2^) for the two equations was 0.95 and 0.94. With no AA supplementation, the estimated intercept and slope of the prediction equations for DE and ME were 14.512 and 20.667, and 14.314 and 19.617 MJ/kg, respectively (p<0.01), and the coefficients of determination (R^2^) for the two equations was 0.94 and 0.93, respectively. Therefore, the DE and ME values of SO determined by these regression equations were 38.02 and 37.17 MJ/kg with AA addition, and 35.18 and 33.93 MJ/kg without AA addition, respectively (on as-fed basis).

The DE and ME values of SO determined by the two methods are compared in Table 6. The average DE or ME value of SO calculated by the difference method were 36.76 and 35.58 MJ/kg with AA addition, and 34.46 and 33.04 MJ/kg without AA addition, respectively (on as-fed basis), which were numerically greater than those acquired by the regression method. However, the values obtained using the difference method with 10% SO substitution fell within the 95% confidence intervals of values obtained using the linear regression method, but these two methods were not equivalent when using 5% SO substitution, with greater values obtained from the regression method.

## DISCUSSION

In this study, we intended to test whether the DE and ME contents of SO were affected by SO inclusion level and AA supplementation in basal diet, and compare the determination methodologies (the difference method vs the regression method). For the dietary parameters tested, the GE output in urine and the urinary N excretion in pigs fed the AA supplementation diets were lower compared with those fed diets without sufficient AA supplementation, despite greater protein intake. These results were not in accordance with the previous literature, which reported that urine N excretion was greater with higher dietary protein intake [15,16]. This phenomenon could be attributed to the unbalanced AA profile in diets when there was not sufficient AA supplementation, which led to the low utilization efficiency of AAs. As a result, addition of crystalline AA is encouraged in swine diets to improve the dietary N retention and reduce N excretion. Compared to the effect of AA addition, the parameters of dietary energy and N balance were more influenced by SO inclusion level, which was consistent with previous studies [15, 16]. As an ingredient with high energy content and energy digestibility, it is reasonable that dietary SO addition can easily facilitate the utilization efficiency of energy. For the other feed ingredients, such as wheat, soybean meal and wheat bran, previous studies showed no differences in GE intake and fecal and urinary GE output among all the treatments, indicating that the energy intake and output were not different as the inclusion level of ingredients changed or without AA addition [16,24]. It’s not consistent with the current research of SO, indicating that the AA profile in these ingredients could cover up the effect of imbalanced AA profile in basal diet when there was no AA supplementation, and the low inclusion level of SO enlarged this effect when calculated using the difference method. Moreover, fecal N excretion decreased with increased inclusion level of SO, which companied with declined dietary CP level. A similar trend was observed in the studies of Su [15] and Jørgensen and Fernandez [18]. The improved ATTD of N agreed with a report that higher inclusion levels of SO improved the digestibility of CP, and may be explained by a reduction in digesta passage rate [25].

The SO inclusion level had a clear effect on the digestibility of GE, DM, OM, and AEE, and the effect was similar to that reported in other studies. The improvement in the nutrient digestibility when lipid was added to pig diets was attributed to a chime flow rate [25]. In addition, the increase in ATTD of AEE with increasing dietary lipid level was also reported when SO [15], corn oil [14] and rapeseed oil [26] were used. On the other hand, AA supplementation of the basal diet had little influence on ATTD of nutrients, which may be due to the higher digestibility of SO. Moreover, in the current study, the diets were formulated with premix containing 100 mg/kg Cu, which had exceeded the suggested supplemental level of copper source in EU (EU Regulation 2018/1039 suggested a copper source level of 25 mg/kg or lower for pigs after 20 kg). The high Cu feeding may lead to the overestimation of the DM and GE digestibility in diets, since previous research reported that the digestibility of DM and GE increased with the addition of Cu in the corn-soy diet [27].

The DE and ME values of SO determined using the difference method were more affected by the SO inclusion level, than AA supplementation of the basal diet, especially for ME value of SO. Although not statistically different, the DE and ME values of SO were numerically higher at the 10% inclusion level compared to the 5% inclusion level, while the previous reports using the corn-soybean meal basal diet showed significant results [15,28]. Furthermore, although not significant, the ME/DE ratio of SO in diets with AA supplementation was numerically greater than that in diets without AA supplementation, which may be due to the lower GE output in urine when AA were added in the basal diet.

In the current study, both the difference and regression method were used to acquire the DE and ME value of SO. The DE and ME values of SO determined with the regression method were similar to the values determined with the difference method when the inclusion level of SO was 10%, and is similar to that reported in other published works [2,9,15, 17]. Actually, in the regression method, the relationship between the energy values (DE or ME) and the inclusion levels of SO were not exactly linear (i.e. R2 was not equal to 1) which is an assumption of the model. This may explain the disparity in DE and ME values of SO between the methods, which was also discussed by Zhao [16] and Su [15]. The DE and ME values of SO were greater when pigs fed AA supplemental diets compared to diets without AA supplementation using both difference and regression methods, which could be explained by the effect of balanced AA profile on energy utilization efficiency.

Some contradictions of SO substitution rate were unavoidable when difference method was used in detection of the DE and ME values. In previous research, Villamide [29] thought a higher standard error would occur with difference method in a low SO substitution rate test diet, though the 5% inclusion level is more important commercially. However, a high substitution rate can improve the accuracy of energy determination, but can cause a nutrient imbalance and influence the nutrient digestibility in diet. Our research found that energy values determined with the difference method at 10% SO substitution were all within the 95% confidence interval of the values determined with the regression method. Similar energy values between the 2 methods were not observed at the 5% SO substitution, indicating that the choice of experimental method in feed evaluation may affect the feed energy value, when low dietary lipid inclusion levels are used (e. g. lower than 5%), so did the dietary crystalline AA supplementation. Although the regression method is commonly used to determine energy value of oil [1,9,12,18], it is time-consuming and costly. Moreover, more experimental animals are required in the regression method to fulfill the graded levels of the test ingredients, increasing the influential factors of the experiment. From the results of the current research, the determined DE and ME values of SO at 10% inclusion level using difference method were similar to those using regression method, thus could act as a substitution for the regression method, but not for SO at 5% inclusion level.

## CONCLUSION

In summary, the DE and ME values of SO are affected by the inclusion level of soybean oil but not AA supplementation. There is no interaction between AA supplementation and SO addition on energy values of SO and dietary nutrient digestibility in the range of 0% to 10%. Therefore, with 5% or less SO addition there should be no concern about the effects of AA supplementation on energy values of SO in actual practice. Moreover, the difference method can substitute the traditionally-used regression method to determine the DE and ME values of SO when the inclusion level is 10%, but not at 5% inclusion level.

## Figures and Tables

**Table 1:** Ingredient and chemical compositions of the control diets used in the experiment (%, as-fed basis)

Items	Control diets1)	

	AA+	AA−
Corn (%)	73.81	74.85
Soybean meal (%)	22.00	22.00
Soybean oil (%)	0.00	0.00
Choline chloride (%)	0.05	0.05
Dicalcium phosphate (%)	1.25	1.25
Calcium carbonate (%)	1.05	1.05
Sodium chloride (%)	0.30	0.30
L-Lys HCl (%)	0.54	0.00
DL-Met (%)	0.15	0.00
L-Thr (%)	0.18	0.00
L-Trp (%)	0.04	0.00
L-Val (%)	0.13	0.00
Vitamin and mineral premix2) (%)	0.50	0.50
Total (%)	100.00	100.00
Calculated compositions		
DE (MJ/kg)	14.05	14.20
ME (MJ/kg)	13.60	13.74
Crude protein (%)	16.47	15.56
Digestible Lys (%	1.09	0.67
Digestible Met:Lys ratio	0.34	0.34
Digestible Met+Cys:Lys ratio	0.55	0.70
Digestible Thr:Lys ratio	0.60	0.72
Digestible Trp:Lys ratio	0.17	0.22
Digestible Val:Lys ratio	0.65	0.88
Ca (%)	0.73	0.73
Digestible P (%)	0.35	0.35

1) AA+, basal diet with amino acid supplementation; AA−, basal diet without amino acid supplementation.

2) Premix provided the following per kg of complete diet for growing pigs: vitamin A, 5,512 IU; vitamin D_3_, 2,200 IU; vitamin E, 30 IU; vitamin K_3_, 2.2 mg; vitamin B_12_, 27.6 μg; riboflavin, 4.0 mg; pantothenic acid, 14.0 mg; niacin, 30.0 mg; choline chloride, 400.0 mg; folacin, 0.7 mg; thiamine 1.5 mg; pyridoxine 3.0 mg; biotin, 44.0 μg; Mn (MnO), 40.0 mg; Fe (FeSO_4_·H_2_O), 75.0 mg; Zn (ZnO), 75.0 mg; Cu (CuSO_4_·5H_2_O), 100.0 mg; I (KI), 0.3 mg; Se (Na_2_SeO_3_), 0.3 mg.

**Table 2:** Analyzed chemical compositions and characteristics of the soybean oil (%, as-fed basis)

Items	Soybean oil
Fatty acids (% of total fat)	
C14:0	0.09
C16:0	11.09
C16:1	0.10
C17:0	0.10
C18:0	4.16
C18:1	22.71
C18:2	52.87
C18:3	6.48
C20:0	0.38
C20:1	0.20
C21:0	0.10
C22:0	0.40
C23:0	0.59
C24:0	0.18
Other fatty acids	0.41
SFA (%)	17.09
UFA (%)	82.37
MUFA	23.01
PUFA (%)	59.35
U:S	4.82
MIU (%)	0.32
FFA (%)	0.10
IV1) (%)	128
Peroxide value (mEq/kg)	1.55
p-Anisidine value (mg/kg)	1.06
Hexanal (mg/kg)	2.65
Total tocopherols (mg/kg)	710

All data are the results of analysis conducted in duplicate.

SFA, saturated fatty acids; UFA, unsaturated fatty acids; MUFA, monounsaturated fatty acids; PUFA, polyunsaturated fatty acids; U:S, the ratio of UFA to SFA; MIU, the sum of moisture, insoluble impurities and unsaponifiable matter; FFA, free fatty acids.

1) IV: iodine value, which was calculated using the fatty acid profile data following the equation proposed by Mireia et al [22]: IV = (C16:1×0.95)+(C18:1×0.86)+ (C18:2×1.732)+(C18:3×2.616)+(C20:1×0.795)+(C20:2×1.57)+ (C20:3×2.38) +(C20:4×3.19)+(C20:5×4.01)+(C22:4×2.93)+(C22:5×3.68)+(C22:6×4.64).

**Table 3:** Effects of soybean oil (SO) inclusion level and amino acid (AA) supplementation on energy content, nutrient digestibility and nitrogen (N) balance of the experimental diets in growing pigs (as-fed basis)

Items	AA addition		SO Inclusion level (%)			SEM	p-value		
		
	AA+	AA−	0	5	10		AA addition	Inclusion level	Interaction
Energy content									
GE (MJ/kg)	17.44	17.58	16.42	17.56	18.56				
GE in feces (MJ/kg)	1.93	2.04	1.96	2.11	1.89	0.09	0.17	0.09	0.96
GE in urine (MJ/kg)	0.18^b^	0.25^a^	0.17^b^	0.24^a^	0.25^a^	0.03	0.01	0.01	0.81
DE (MJ/kg)	15.50	15.55	14.45^c^	15.46^b^	16.68^a^	0.09	0.59	<0.01	0.25
ME (MJ/kg)	15.32	15.30	14.29^c^	15.22^b^	16.43^a^	0.09	0.75	<0.01	0.21
ME/DE (%)	98.83^a^	98.40^b^	98.86	98.48	98.52	0.17	<0.01	0.07	0.84
ATTD (%)									
GE	88.52	88.37	87.51^c^	88.04^b^	89.60^a^	0.25	0.27	<0.01	0.93
DM	89.72	90.15	89.09^b^	90.28^a^	90.44^a^	0.16	0.10	<0.01	0.21
OM	91.08	91.70	90.37^b^	91.91^a^	91.89^a^	0.15	0.11	<0.01	0.19
AEE	77.42	75.42	60.90^c^	81.26^b^	87.10^a^	0.41	0.09	<0.01	0.24
N balance (g/kg BW^0.75^/24 h)									
N intake	26.88	25.96	27.81	26.42	25.03				
Fecal N excretion	3.47	3.47	3.63^a^	3.82^a^	2.98^b^	0.09	0.66	0.04	0.42
Urinary N excretion	5.68^b^	8.52^a^	6.55	7.21	7.55	0.30	0.02	0.62	0.12
N retention	17.72^a^	13.97^b^	17.64^a^	15.40^b^	14.51^c^	0.32	0.04	0.04	0.18
ATTD of N	87.09	86.65	86.96^b^	85.56^c^	88.10^a^	0.21	0.19	0.04	0.21

Data are means of six observations.

SEM, standard error of the mean; GE, gross energy; DE, digestible energy; ME, metabolizable energy; ATTD, apparent total tract digestibility; DM, dry matter; OM, organic matter; AEE, acid-hydrolyzed ether extract; BW, body weight.

a–c Means within a row without a common lower-case letter differs significantly (p<0.05).

**Table 4:** Energy content of soybean oil (SO) in growing pigs determined by the difference method (as-fed basis)

Items	AA addition		SO inclusion level (%)		SEM	p-value		
		
	AA+	AA−	5	10		AA addition	Inclusion level	Interaction
DE (MJ/kg)	36.76	34.46	34.57	36.65	-	0.09	0.12	0.65
ME (MJ/kg)	35.58	33.04	33.01	35.61	0.73	0.07	0.05	0.53
ME/DE (%)	96.73	95.83	95.42	97.13	0.59	0.46	0.17	0.78

Data are means of six observations.

SEM, standard error of the mean; DE, digestible energy; ME, metabolizable energy; AA, amino acid.

**Table 5:** Energy content of soybean oil (SO) in growing pigs determined by the regression method (as-fed basis)

Items	Regression equations1)	R^2^	RMSE	Intercept		Slope		When x = 1, estimation of DE or ME
	
				SEM	p-value	SEM	p-value	
AA+								
DE	y = 14.320+23.700x	0.95	0.22	0.08	<0.01	1.27	<0.01	38.02
ME	y = 14.169+23.000x	0.94	0.24	0.09	<0.01	1.38	<0.01	37.17
AA−								
DE	y = 14.512+20.667x	0.94	0.22	0.08	<0.01	1.29	<0.01	35.18
ME	y = 14.314+19.617x	0.93	0.23	0.09	<0.01	1.35	<0.01	33.93

RMSE, root of the mean square error; SEM, standard error of the mean; AA, amino acid; DE, digestible energy; ME, metabolizable energy.

1) In equation y = ax+b, y = DE or ME values of diet (MJ/kg), x = inclusion level of soybean oil.

**Table 6:** Comparison of the energy content (MJ/kg) of soybean oil in growing pigs determined by difference method and regression method with 5% or 10% soybean oil (SO) included in diets (as-fed basis)

Items	Difference method with 5% dietary SO inclusion		Difference method with 10% dietary SO inclusion		Regression method		95% Confidence interval from the regression method	
			
	AA+	AA−	AA+	AA−	AA+	AA−	AA+	AA+
DE	35.42	33.71	38.09	35.21	38.02	35.18	35.41–40.63	32.53–37.82
ME	33.91	32.10	37.25	33.97	37.17	33.93	34.33–40.00	31.16–36.70

AA, amino acid; DE, digestible energy; ME, metabolizable energy.
